# Effect of Intramuscular Medetomidine Administration on Tear Flow in Rats

**DOI:** 10.3390/vetsci7020042

**Published:** 2020-04-13

**Authors:** Teppei Kanda, Yuka Mizoguchi, Kayo Furumoto, Yuki Shimizu, Noritaka Maeta, Toshinori Furukawa

**Affiliations:** 1 Faculty of Veterinary Medicine, Okayama University of Science, 1-3 Ikoino-oka, Imabari, Ehime 794-8555, Japan; 2 Department of Comparative Animal Science, College of Life Science, Kurashiki University of Science and the Arts, 2640 Nishinoura, Tsurajima-cho, Kurashiki, Okayama 712-8505, Japan; 3 Department of Animal Pharmaceutical Science, School of Pharmaceutical Science, Kyushu University of Health and Welfare, 1714-1 Yoshino-cho, Nobeoka, Miyazaki 882-8508, Japan

**Keywords:** α_2_-adrenoceptor agonist, medetomidine, tear flow, phenol red thread test, rat

## Abstract

Medetomidine has been reported to decrease tear flow significantly in dogs, cats, and pigs when used as a sedative or analgesic; however, there are no such reports when it comes to rats. The present study aimed to investigate the effect of medetomidine on tear flow in rats. Medetomidine in doses of 50, 100, or 200 µg/kg or a physiological saline solution as the control, were administered intramuscularly to male Slc:Wistar/ST rats. After the administration of medetomidine, tear flow in both eyes was measured using a phenol red thread tear test. The area under the curve (AUC) of phenol red thread test values from baseline to 8 h was calculated. Data were plotted against the dose of medetomidine and simple linear regression analysis was performed. The effect of the drug on phenol red thread test values was considered dose-related when linear analysis yielded a significant relationship. In all medetomidine-treated groups, tear flow decreased significantly in both eyes after administration, while no significant changes were observed in either eye in the control group. The AUC values from baseline to 8 h after administration in groups treated with 100 and 200 µg/kg of medetomidine were significantly lower in both the left and right eyes compared to the control group. The linear regression of the AUC values was significant for both eyes. Our results indicated that the intramuscular administration of medetomidine in rats decreased tear flow significantly in a dose-dependent manner.

## 1. Introduction

Medetomidine, an α_2_-adrenoceptor agonist, is widely used as a sedative or analgesic in veterinary medicine [1,2,3,4,5,6,7,8]. In addition to medetomidine, other α_2_-adrenoceptor agonists such as detomidine [9,10,11], dexmedetomidine [1,12,13,14], xylazine [4,7,9,14,15], and romifidine [9,10,16,17,18,19,20] are also used clinically in many domestic species. Furthermore, some α_2_-adrenoceptor agonists, including medetomidine, are administered not only alone, but also in combination with other drugs, such as opioids, benzodiazepine, phenothiazine, and ketamine [3,5,6,8,21,22,23,24,25,26,27]. Although medetomidine and the other α_2_-adrenoceptor agonists used alone or in combination with other drugs have potent sedative or analgesic effects, they have been reported to significantly decrease tear flow in dogs [28,29,30,31,32], cats [33,34], horses [35,36], and pigs [37].

Tear fluids play an essential role in maintaining the immune system of the ocular surface and the metabolic processes of the cornea, which is an avascular tissue. Additionally, they maintain the smoothness of the optical surface by lubricating the cornea, conjunctiva, and nictitating membrane [38]. Therefore, an insufficient tear flow could cause pain or irritation on the ocular surface and could result in a vision disorder. A recent study also reported that tear fluid deficiency causes hypersensitivity in the rat cornea [39]. A small stimulus is likely to induce great irritation or pain even though there is no physical damage to the cornea. Apparently, it negatively affects the welfare of animals. Tear fluid deficiency should be the focus of current research, not only in order to protect ocular tissue and function, but also to relieve the irritation or pain.

In rats, a representative laboratory animal, medetomidine is administered both alone and in combination with other drugs [40,41,42,43,44,45,46]. However, the effect of medetomidine on tear flow has not been investigated, although clonidine, one of the α_2_-adrenoceptor agonists, was reported to decrease tear flow [47]. In horses, xylazine [48] and romifidine [36] were reported not to decrease tear flow, although detomidine was found to decrease it significantly [36]. This means that even if the administered agent is one of the α_2_-adrenoceptor agonists, its ability to decrease tear flow in a specific animal species varies with each drug. Therefore, the result of the study with clonidine might not necessarily be replicated in rats administered medetomidine. It is essential to know whether tear flow decreases in rats administered medetomidine to avoid inducing pain or irritation on the ocular surface, which could affect their welfare. 

In the present study, we aimed to investigate the effect of medetomidine on tear flow in rats.

## 2. Materials and Methods

### 2.1. Animals

Male Slc:Wistar/ST rats (*Rattus norvegicus*) (n = 24, aged 8–11 weeks; Japan SLC, Inc., Hamamatsu, Japan) weighing 325 ± 23 (mean ± standard deviation (SD)) g were used. The animals were allowed to acclimatize for a week before treatment and were housed in groups of two to three animals in individually ventilated cages (NIKI SHOUJI Co., Tokyo, Japan) placed in one room. A 12:12 h light-dark cycle (light period, 8:00 a.m. to 20:00 p.m.) was maintained, room temperature was kept between 24 and 26 °C, and humidity was at 40–60%. Water and a commercially pelleted diet (CE-2; CLEA Japan, Inc., Tokyo, Japan) were provided ad libitum. After the intramuscular administration of medetomidine or saline, a single rat was placed in an individually ventilated cage on the laboratory table of the experimental room. The rat stayed there for 24 h between measurements. Wood shavings (CL-4161; CLEA Japan, Inc., Tokyo, Japan) were used as bedding material in all cages. Six rats were assigned randomly to any of the four treatment groups and were not blinded. All procedures performed were approved by the Animal Care and Use Committee of Kurashiki University of Science and the Arts (approval number, 25-12).

### 2.2. Drug Treatment

Medetomidine (1000 µg/mL, Domitor; Nippon Zenyaku Kogyo Co., Ltd., Fukushima, Japan) in doses of 50, 100, or 200 µg/kg, or a physiological saline solution (0.2 mL/kg) as the control, were administered intramuscularly to the rats. According to the treatment received, groups were named MED50, MED100, MED200, and control group, respectively. The drug solution was injected into the caudal part of the left thigh using a micro syringe (1/2 mL BD Lo-Dose Insulin Syringe 29 G × 1/2 inch; BD, Franklin Lakes, NJ, USA) at 10:00 a.m.

### 2.3. Measurement

Tear flow was measured using a phenol red thread tear test (Zone-Quick; AYUMI Pharmaceutical Corporation, Tokyo, Japan) in both eyes [49,50]. The 3 mm tips of threads were placed on the medial canthus for 15 s. The length of the moistened area from the edge was measured as the phenol red thread test (PRTT) value. Tear flow was measured approximately 1 min before and 0.25, 0.5, 1, 2, 3, 4, 5, 6, 7, 8, and 24 h after medetomidine administration.

### 2.4. Observation

The state of the ocular surface, including corneal opacity, was observed grossly, together with recording the measurements of PRTT values. The presence of any significant side effects was monitored throughout the experiments.

### 2.5. Statistical Analysis

The Wilcoxon matched-pairs signed-rank test was used to examine the differences in PRTT values between the left and right eyes at each time point for each treatment. The time effect on each treatment was evaluated using the Friedman test for repeated measures. When a significant change was found, Dunn’s multiple comparison test was used to compare the mean value at each time point with the baseline value in each treatment. The area under the curve (AUC) of PRTT values from baseline to 8 h was calculated. Data were plotted against the dose of medetomidine and simple linear regression analysis was performed. The effect of a drug on PRTT values was considered dose-related when linear analysis yielded a significant relationship. The Kruskal–Wallis test was used for comparison of mean AUC values. When a significant change was found, Dunn’s multiple comparison test was used to compare the mean AUC values between treatments. PRTT and AUC values were reported as mean ± standard error of the mean (SEM) and mean ± standard deviation (SD), respectively. Mean values of age and body weight in each group were compared using the one-way analysis of variance (ANOVA). When a significant difference was found, Tukey’s multiple comparison test was used to compare the mean values between groups. All statistical analyses were performed using GraphPad Prism 8 (GraphPad Software, San Diego, CA, USA). A *p*-value < 0.05 was considered statistically significant.

## 3. Results

There were no statistical differences in age and body weight between the groups.

**Figure 1:**
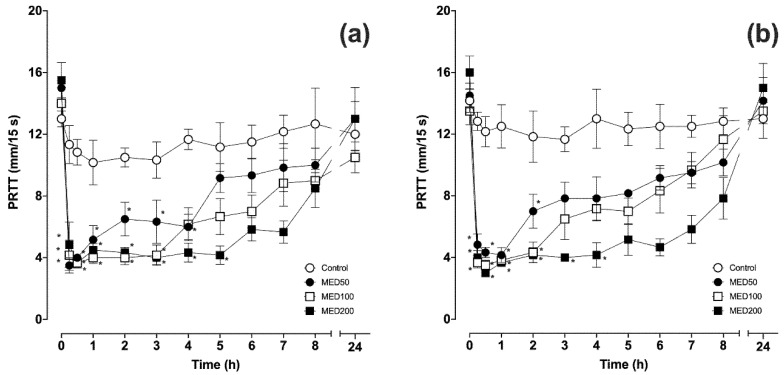
Mean ± standard error of the mean (SEM) of phenol red thread test (PRTT) values in the left (**a**) and right (**b**) eyes before and after intramuscular administration of medetomidine (n = 6). * *p* < 0.05 compared with the baseline value.

The baseline PRTT values in the left and right eyes were 13.0 ± 0.5 and 14.2 ± 0.7 mm/15 s respectively, in the control group, 15.0 ± 0.7 and 14.5 ± 0.8 mm/15 s respectively, in MED50, 14.0 ± 1.0 and 13.5 ± 0.9 mm/15 s respectively, in MED100, and 15.5 ± 1.1 and 16.0 ± 1.0 mm/15 s respectively, in MED200. There were no differences in PRTT values between the left and right eyes in each time point for all groups. In the control group, there were no significant changes in either eye (Figure 1). In MED50, PRTT values decreased significantly 0.25–4 h (3.5 ± 0.5, 4.0 ± 0.3, 5.2 ± 0.9, 6.5 ± 1.1, 6.3 ± 1.4, and 6.0 ± 0.8 mm/15 s, respectively) and 0.25–2 h (4.8 ± 0.7, 4.3 ± 0.3, 4.2 ± 0.5, and 7.0 ± 1.1 mm/15 s, respectively) after the intramuscular administration of medetomidine in the left and right eye, respectively. These decreases were followed by a gradual recovery to the baseline value. In MED100, significant decreases in PRTT values were observed 0.25–3 h (4.2 ± 1.0, 3.7 ± 0.3, 4.0 ± 0.4, 4.0 ± 0.5, and 4.2 ± 0.3 mm/15 s, respectively) and 0.25–2 h (3.7 ± 0.3, 3.5 ± 0.3, 3.8 ± 0.2, and 4.3 ± 0.7 mm/15 s, respectively) after the administration of medetomidine in the left and right eye, respectively. In MED200, significant decreases in PRTT values were observed 0.25–5 (4.8 ± 1.2, 3.7 ± 0.3, 4.5 ± 0.5, 4.3 ± 0.3, 4.0 ± 0.5, 4.3 ± 0.6, and 42. ± 0.6 mm/15 s, respectively) and 0.25–4 h (4.0 ± 0.4, 3.0 ± 0.0, 3.7 ± 0.2, 4.2 ± 0.5, 4.0 ± 0.3, and 4.2 ± 0.8 mm/15 s, respectively) after the administration of medetomidine in the left and right eye, respectively. PRTT values remained at lower levels for longer in MED200 than they did in MED50 and MED100. Twenty-four hours after the intramuscular administration of medetomidine, the PRTT values of both eyes returned to the baseline levels in all treatment groups. 

Throughout the experiments, in all groups, no gross changes were observed in the ocular surfaces, including the cornea. In addition, no significant side effects related to the α_2_-adrenoceptor agonists, such as severe cardiopulmonary depressions or neurological abnormalities, were observed throughout the experiments.

**Figure 2:**
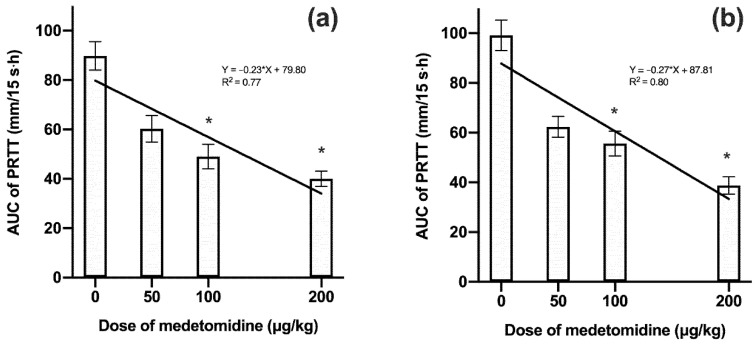
Mean ± standard deviation (SD) of area under the curve (AUC) values in the left (**a**) and right (**b**) eyes during the 8 h period after the intramuscular administration of various doses of medetomidine (n = 6). * *p* < 0.05 compared with the control group.

The AUC values from baseline to 8 h after administration in MED100 and MED200 were significantly lower in both the left and right eyes compared to the control group. The linear regression of the AUC values was significant for both eyes (Figure 2).

## 4. Discussion

The results of the present study indicated that the intramuscular administration of medetomidine significantly decreased tear flows in rats. The reference range of PRTT values in different rat strains, or rats in general for that matter, has not been reported. In previous studies, the control PRTT values in Sprague-Dawley rats were reported as 10 ± 1.0 mm/15 s for first-time measurements and 10.8 ± 1.9 mm/15 s for second-time measurements (8 weeks after the first measurements and vehicle administration) by Hegarty et al. [49], or approximately 13 mm/15 s by Nakamura et al. (shown in a graph without values) [50]. In this study, similar PRTT values were obtained for the control group, although the strain was different. Based on these facts, it could be suggested that the decrease in tear flow after intramuscular administration of medetomidine was significantly below the physiological range for rats.

The higher the dose of medetomidine, the longer the tear flow decrease lasted compared to the baseline value, while the lowest tear flow value was similar in all groups treated with medetomidine. AUC values from baseline to 8 h after administration indicated that the decrease of tear flow in rat was dose-dependent. Therefore, it is advisable to reduce the dose of medetomidine to avoid severe decrease in tear flow due to the high doses.

It was reported that α_2_-adrenoceptor agonists, including medetomidine, decrease tear flow in many species, such as dogs, cats, horses, and pigs [28,29,30,31,32,33,34,35,36,37]. The mechanism through which α_2_-adrenoceptor agonists decrease tear flow is not yet completely clarified, although local or systemic involvement of an α_2_-adrenoceptor was suggested in previous studies [28,31,32,34,36]. Some mechanisms through which α_2_-adrenoceptor agonists, including medetomidine, possibly decrease tear flow have been previously reported [31,32,34,36]. Among these, the neurophysiological mechanisms and hemodynamic change involved with the α_2_-adrenoceptor have been discussed as the most likely reasons [32,36]. Leonardi suggested that postsynaptic activation of α_2_-adrenoceptors in the central nervous system due to α_2_-adrenoceptor agonists might have decreased basal tear production in previous studies on dogs and horses [31,36]. The fact that atipamezole, an α_2_-adrenoceptor antagonist, recovered or attenuated the decrease in tear flow induced by α_2_-adrenoceptor agonists indirectly supports these suggestions [28,31]. Although adrenergic nerve fibers innervate the lacrimal glands in mice and dogs [51,52], this has not been identified in rats. Medetomidine induces systemic vasoconstriction, lower heart rate, and a decrease in cardiac output in mammals [53,54]. Consequently, perfusion of the lacrimal glands should decrease after medetomidine administration. Such lowered perfusion of lacrimal glands has been suggested to be responsible for α_2_-adrenoceptor agonist-induced decrease in tear flow [31,34,36]. As another possible mechanism, alteration of metabolism at the lacrimal gland cellular level through the α_2_-adrenoceptor has been proposed to decrease tear production [32]. At present, although the existence or function of α_1_-adrenoceptor in the lacrimal gland in rats or mice has been reported, there have been no reports regarding the α_2_-adrenoceptor in the lacrimal glands of any species [55,56]. Furthermore, it was also suggested that increased antinociception modulated by α_2_-adrenoceptor decreases reflex tear secretion [32].

In this study, no significant gross corneal lesions were observed in rats even though tear flow decreased significantly. Therefore, we concluded that the medetomidine-induced decrease in tear flow did not cause any gross corneal opacity lesions in rats at the tested doses of medetomidine.

There were several limitations in this study. We could not discuss the relationship between the effects of medetomidine on the central nervous system, such as sedation or changes in autonomic tone, and decrease in tear flow, because the degree and duration of sedation and physiological parameters, including heart rate and respiratory rate, were not measured. Additionally, we did not measure the PRTT values between 8 to 24 h after medetomidine administration; thus, we could not determine how long the decrease in PRTT persisted.

## 5. Conclusions

We concluded that the intramuscular administration of medetomidine led to a dose-dependent and significant decrease in tear flow in rats, measured by PRTT. Although the decrease in tear flow induced by medetomidine did not cause a gross corneal lesion within 24 h after administration, the ocular surface should be treated to protect ocular health and the welfare of the rats.
